# *In vitro* effects of velvet antler water extracts from Formosan Sambar deer and red deer on barrier integrity in Caco-2 cell

**DOI:** 10.7150/ijms.53599

**Published:** 2021-02-18

**Authors:** Ying-Kai Hung, Shang-Tse Ho, Ching-Yun Kuo, Ming-Ju Chen

**Affiliations:** 1 Department of Animal Science and Technology, National Taiwan University, Taipei 106, Taiwan.; 2 Department of Wood Based Materials and Design, National Chiayi University, Chiayi 600, Taiwan.; 3 Taiwan Livestock Research Institute, Council of Agriculture, Tainan 712, Taiwan.

**Keywords:** Caco-2 cell model, tight junction proteins, velvet antler.

## Abstract

**Background:** The mucus integrity and abnormal inflammatory response are the crucial biomarker of inflammatory bowel disease (IBD). Velvet antler (VA) has been used as traditional Chinese medicines for many years. Anti-inflammatory property was demonstrated *via* suppression of cyclooxygenase-2 and cytokines protein expression. And it has further proved to promote wound healing in streptozotocin-induced diabetic rats model. The aforementioned functionalities of VA extracts may be associated with the treatment of IBD. Thus, the aim of present study was to evaluate the effect of velvet antler water extracts form Formosan Sambar deer (*Rusa unicolor swinhoei*, SVAE) and red deer (*Cervus elaphus*, RVAE) on the barrier function and to investigate the possible mechanism using *in vitro* model.

**Methods:** Human colonic epithelial cell models (Caco-2) were co-cultured with various concentrations of both SVAE and RVAE (250-500 µg mL^-1^) in dextran sulfate sodium (DSS)-induced colitis model. Trans-epithelial electrical resistance (TEER) value and the macromolecule permeability of Fluorescein isothiocyanate (FITC)-labeled dextran were measured to evaluate the integrity of monolayer of Caco-2. Western blotting was performed for analysis of protein expressions of occludin, Zonula occludens-1 (ZO-1), claudin-1, claudin-2 and myosin light chain kinase (MLCK). The cytotoxicity was conducted by MTT assay.

**Results:** Results indicated that both SVAE and RVAE could enhance integrity of monolayer in dextran sulfate sodium (DSS)-induced colonic epithelial cell model (Caco-2) through reducing the decline of trans-epithelial electrical resistance (TEER) and macromolecule permeability at the concentration of 250 μg mL^-1^. RVAE significantly increased the expression of tight junction proteins (occludin and ZO-1) while SVAE significantly reduced the activity of MLCK (*P* < 0.05.). Elevated C-C chemokine ligand 20 (CCL20) production suggested that both SVAE and RVAE could enhance the repair of epithelial cell. Besides, MTT assay revealed that both extracts showed no cytotoxicity.

**Conclusion:** Thus, SVAE and RVAE supplementation may attenuate barrier damage by enhancing the occludin and ZO-1 protein expression, decreasing MLCK expression, promoting the CCL20 production. In the future, animal study is needed for further confirmation.

## Introduction

Velvet antler (VA), cartilaginous antler that has not been fully calcified, is one of the most famous and valuable traditional Chinese medicines. From the previous studies, various pharmacological properties were confirmed, including immunomodulatory, anti-fatigue, anti-osteoporosis and tissue repair effects with different extraction methods 1, 2. Anti-inflammatory property of VA was demonstrated *via* suppression of nitrogen oxide production and the cyclooxygenase-2 protein expression *in vitro*, while its pro-inflammatory cytokines were down-regulated in allergic asthma model 3, 4. Besides, several researches indicated that VA comprises certain growth factors that relating to its annual regeneration, and might aid proliferation of epidermal cell and wound healing 5-8. The aforementioned functionalities of VA extracts may be associated with the treatment of inflammatory bowel disease (IBD).

IBD, including ulcerative colitis and Crohn's disease, are characterized by chronic debilitating inflammation of gastrointestinal tract and epithelial lesions. In the past decades, the prevalence and incidence of IBD are gradually increasing in the world 9, 10. Due to the complicated interaction between host immune system and homeostasis with gut microbiota, the etiology remains unknown. Current therapies are corticosteroids, monoclonal antibody and antibiotics which target on pro-inflammatory cytokines of the aberrant inflammatory response and some pathogen individually. However, the efficacies of current therapeutic agents is sometimes limited and then lead to the colitis relapsing with several side effects 11, 12. In addition to inflammation, disruption of mucus epithelial is one of the features of IBD as well. Emerging studies have revealed that tight junction proteins, regulating the permeability of luminal fluid, are crucial in maintaining the integrity of intestinal mucus 13-16.

Tight junction proteins, such as occludin and claudin family, are important members that contribute to the function of physical barrier at the end of the gastrointestinal lumen 17. These transmembrane proteins are further anchored by zonula-occludens (ZO) families which are linked to the cytoskeleton protein in cell. The phosphorylation of myosin light chain kinase (MLCK) plays a vital role to activate the mobilization of those proteins 18,19. Previous research reported that epithelial MLCK is critical to barrier dysfunction *via* tumor necrosis factor receptor-2's pathway in mice colitis model 20. In addition, both pro-inflammatory cytokine of tumor necrosis factor-α (TNF-α) and interferon-γ (IFN-γ) would upregulate the expression of MLCK, which are relevant to systematic inflammation 21-23. In previous findings, VA extracts could alleviate the symptoms of inflammatory response in collagen-induced arthritis model and ovalbumin-allergic asthma model 24-25, demonstrating the potential for intestinal protection.

Although velvet antler has been consumed for thousand years, there are no investigations on the influence of VA on colitis. In our previous study, Formosan Sambar VA water extracts could protect against cell infection by down-regulation of pro-inflammatory cytokines (TNF-α and interleukin-6 (IL-6)) and reduction of phagocytosis 3, 26. Whereas, Red VA extracts is proved to promote wound healing in streptozotocin-induced diabetic rats model 5,7. Taken together, anti-inflammatory and cell proliferation properties of VA might be applied to recover the mucosal barrier function of colitis. Thus, the aim of present study was to evaluate the effects of VA water extracts from Taiwanese indigenous Formosan Sambar deer and red deer to against DSS-induced colitis model *in vitro*. The possible mechanism of VA on mucosal integrity is also investigated.

## Materials and Methods

### Chemicals and reagents

3-(4,5-Dimethylthiazol-2-yl)-2,5-diphenyltetrazolium bromide (MTT), dimethyl sulfoxide (DMSO), Fluorescein isothiocyanate (FITC)-labeled dextran (average molecular weight: 10,000 Da), human holo-transferrin and Lipopolysaccharide (LPS) were purchased from SigmaeAldrich (St. Louis, MO, USA). Dextran sulfate sodium (DSS) was purchased from MP BioChemicals (molecular weight: 36,000-50,000 Da; Santa Ana, CA, USA). DMEM (Dulbecco's Modified Eagle Medium), fetal bovine serum (FBS), and other cell culture reagents were obtained from Corning (Tewksbury, MA, USA). All of the chemicals and solvents used in this research were of analytical grade.

### Preparation of velvet antler water extracts

VA samples from Formosan Sambar deer were reared between 65-70 days and kindly provided from Kaohsiung Animal Propagation Station, Taiwan Live Stock Research Institute (Pintong, Taiwan). VA samples from red deer were reared within 70-75 days and purchased from Feng Ying Deer Ranch (Tainan, Taiwan). Fresh VA samples were immediately sliced and stored at -80°C. The frozen VA slices were first dehydrated by freeze dryer (Kingmech Co. Ltd., Taipei, Taiwan) and ground into powder. The VA powder was extracted by soaking at 4°C water (50 g L^-1^) in Ultrasonic Cleaner (Delta, Co. Ltd., Taipei, Taiwan) for an hour, then cooled for 15 minutes. These procedures were repeated for twice. The supernatants were then collected and dehydrated by lyophilization to harvest Formosan Sambar velvet water extracts (SVAE) and red velvet water extracts (RVAE).

### Caco-2 cell culture

Human colonic epithelial cell line Caco2-C2BBe1 was purchased from Bioresource Collection and Research Center (BCRC, Hsinchu, Taiwan) and cultured in DMEM containing 10% heat-inactivated FBS, 0.1% human holo-transferrin, 1% antibiotic antimycotic and 1% sodium pyruvate in a humidified incubator with 5% CO_2_ at 37°C. The cells were sub-cultured at a density of 2 × 10^5^ cells/flask in a 75 cm^2^ flask between 5 to 6 days.

### Cytotoxicity test

The cytotoxic assays were performed and modified as described 27. Caco-2 cells were seeded onto 48-well plates at a density of 1× 10^4^ cells/well overnight. After overnight incubation, the medium was replaced with DMEM containing VA samples (125, 250 and 500 μg mL^-1^) or DSS (3%), and followed another 48 hours of co-culture. The cells of control group were cultured using DMEM alone. After incubation, the supernatant was removed and the cells were washed with PBS twice. Then, 200 μL of MTT solution (1 mg mL^-1^ in DMEM) was added and incubate for 3.5 hours. Subsequently, the solution was discarded and the residues were dissolved in 200 μL DMSO. Absorbance at 540 nm was measured by ELISA reader (Epoch, BioTek Instruments, Inc., VT, USA). Recorded values were normalized with control group.

### Preparation of Caco-2 cell monolayer

Caco-2 cells were seeded onto permeable 12-well transwell plate (Corning, Lowell, MA, USA) with a 3 μm pore size at a density of 1.5 x 10^5^ cells/well to form the epithelial monolayer. Both the apical and basolateral sites of Transwell were replaced with fresh medium every 2 days, while the trans-epithelial electrical resistance (TEER) value was measured using EVOM epithelial voltohmmeter coupled with an STX2 probe (World Precision Instruments, Sarasota, FL, USA). The Caco-2 monolayers were ready to use when the TEER values were greater than 500 Ω/cm^2^ with cultivation more than 21 days 28, 29.

### Assessment of integrity of epithelial monolayer with DSS-induced damage

Integrity of Caco-2 cell monolayer was evaluated using TEER measurement and FITC-dextran permeability assay. VA samples (250 and 500 μg mL^-1^ in DMEM) were added continuously in the apical site of transwell plate for 21 days until the monolayer was formed. Then, the medium was discarded and replaced with the fresh medium containing same dosage of VA samples and 3% DSS. The TEER value were monitored for 24 hours. In terms of permeability assay, the monolayer of Caco-2 cells was cultured for 14 days with indicated dosage of VA samples. After cultivation for 14 days, the medium was replaced with medium containing 3% DSS and then cultured for 48 hours. Two mg mL^-1^ FITC-label dextran of medium was added in the apical site of transwell plate. After 2 hours-incubation, the medium in the basolateral site were collected and the absorbance of fluorescence at 485 and 528 nm wavelength were quantified 28, 30.

### Western blot analysis

After formation of epithelial monolayer of Caco-2 cell, the supernatant was discarded. Cell debris was scraped and lysed in 100 μL of radioimmunoprecipitation assay (RIPA) buffer containing 1% protease inhibitor and 1% 0.5 M EDTA (Sigma-Aldrich, St. Louis, MO, USA), then boiled in a water bath at 100°C for 10 min. The protein supernatant was collected by centrifugation at 12,000 × *g* for 30 min and then quantified by the bicinchoninic acid (BCA) protein assay kit (Thermo Fisher Scientific, Waltham, MA, USA). Five μg of protein samples were loaded into 8%, 10% and 12% sodium dodecyl sulfate polyacrylamide gel respectively for protein analysis of Zonula occluden-1, Occludin and claudin, respectively. After electrophoresis, proteins were transferred to a polyvinylidene fluoride (PVDF) membrane (90 min), and soaked in blocking buffer (Thermo Fisher Scientific, Waltham, MA, USA) for 15 min. The membranes were incubated with primary antibodies of Claudin-1 (ab15098), Claudin-2 (ab53032), Occludin (ab216327), ZO-1 (ab96587), MLCK (ab76092) and β-actin (ab16039) (Abcam, Cambridge, MA, USA) at 4°C overnight. The membrane was washed for 3 times with TBS-Tween buffer, and incubated in horseradish peroxidase (HRP)-labeled rabbit secondary antibodies (Abcam, Cambridge, MA, USA) for an hour. After incubation, membranes were immersed with western lighting ECL pro reagent (Perkin-Elmer, Waltham, MA, USA) and were analyzed by the ChemiDoc Touch Imaging System (Bio-Rad, Hercules, CA, USA). The quantification of protein expression was performed by Image J 31.

### Measurement of CCL20 production

Caco-2 cells were seeded onto 48-well plate at a density of 1.0 x 10^6^ cells/well for overnight. The medium was replaced with DMEM containing VA samples (62.5 125, 250 and 500 μg mL^-1^) or DSS (3%) for 24 hours. After incubation, the supernatants were collected by centrifugation at 1,500 × *g* for 10 min and were further measured by DuoSet ELISA Development Systems (R&D Systems, Minneapolis, MN, USA) according to the manufacturer's instructions 32.

### Statistics

Values were given as mean ± standard deviation. All results were analyzed by Student's t-test compared with the negative control group (NC); *P* < 0.05 was considered as statistically significant. All experiments were performed in triplicates.

## Results

### Velvet antler water extracts showed no cell cytotoxicity

**Figure 1:**
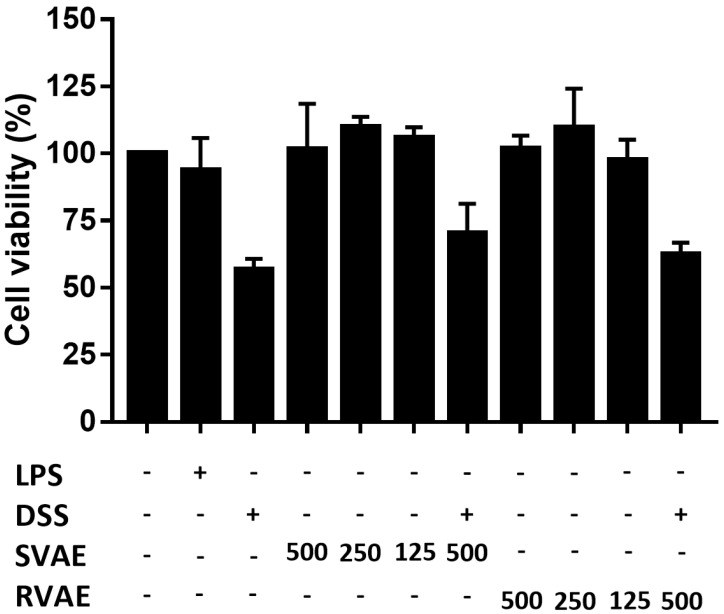
Effects of VA water extracts and their concentration on cell viability of Caco-2 cells. The values are mean ± SD (n = 3). The samples at different dosages of VA water extracts are added and co-culture for 48 hours. After treatment, the medium is removed and the cells were washed. Cell viability is measured by MTT assay. DSS: dextran sodium sulfate. SVAE: Sambar velvet antler water extracts. RVAE: red velvet antler water extracts. LPS: Lipopolysaccharide.

Firstly, to evaluate the cytotoxicity of VA water extracts on Caco-2 cells, the effects of VA water extracts on cell viability were determined by MTT assay *in vitro*. As shown in Figure 1, both SVAE and RVAE showed no cytotoxic effect compared to the control group at concentrations ranging from 125 to 500 μg mL^-1^. Cell viability was dropped to 56.82% when subjected to DSS injury on Caco-2 cells, while addition of VA extracts could enhance the cell viability to the range of 62.56-70.23%.

### Velvet antler water extracts enhanced the barrier function of Caco-2 monolayer against DSS challenge

**Figure 2:**
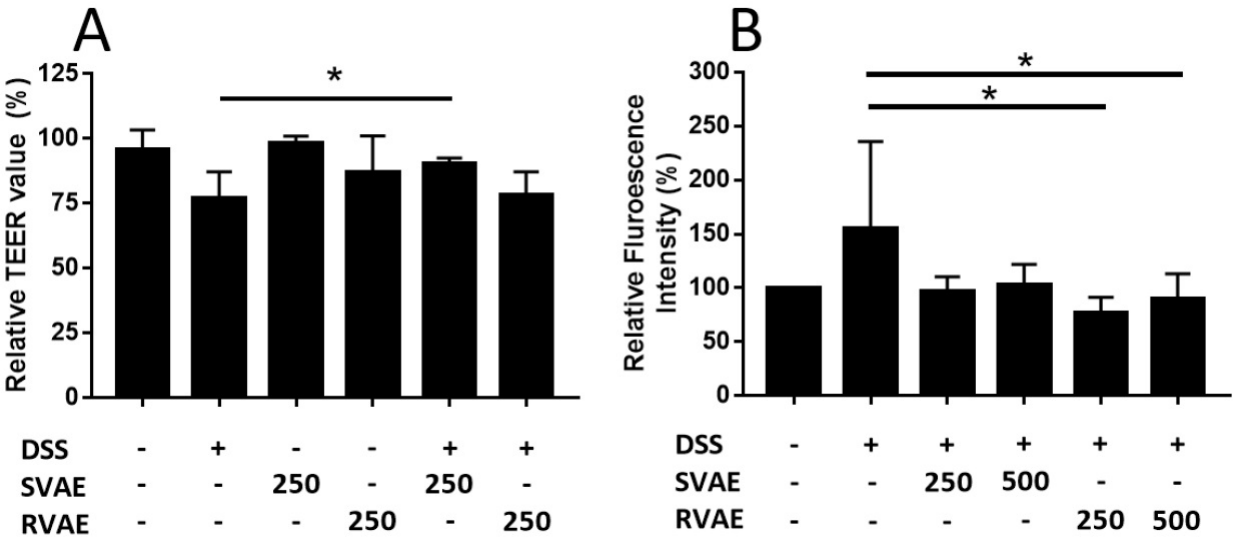
Effects of VA water extracts on barrier function of monolayer integrity by (A) transepithelial electric resistance (TEER) value and (B) permeability of fluorescein isothiocyanate-labeled dextran in Caco-2 cell. Values are mean ± SD (n = 6 of (A) and n = 8 of (B)). * Significantly difference (*P* < 0.05) was compared with the DSS-treated group (negative control). Both SVAE and RVAE samples are co-culture with Caco-2 cells at the dosage of 250 μg mL^-1^ and 500 μg mL^-1^. DSS is added to the medium and monitored for the change of TEER value at 6 hours. Permeability test of fluorescein isothiocyanate-labeled dextran is added in the apical site and measures in the basolateral site after 2 hours. DSS: dextran sodium sulfate. SVAE: Sambar velvet antler water extracts. RVAE: red velvet antler water extracts.

The epithelial barrier function of Caco-2 monolayer was evaluated by the assay of TEER value measurement and permeability test of FITC-dextran. In TEER detection, the value was decreased after treated with DSS for 6-hours cultivation, while the SVAE treatment group (250 μg mL^-1^ extracts) showed a significantly enhancement (*P* < 0.05) (Figure 2A). We further monitored the molecule permeability of FITC-dextran through detecting fluorescence intensity at basolateral site of the Caco-2 monolayer. As the Caco-2 monolayer exposed to DSS, the fluorescence intensity was increased obviously after 48 hours. Co-culturing with VA extracts at the dosage of both 250 and 500 μg mL^-1^ showed lower permeability compared to the DSS-treated group, implying that minor degree of leakage of the Caco-2 monolayer, especially in RVAE group (*P* < 0.05) (Figure 2B). These findings indicated that both SVAE and RVAE are potential to protect the epithelial monolayer against DSS damage.

### Velvet antler water extracts protected the tight junction associated proteins of Caco-2 cells

**Figure 3:**
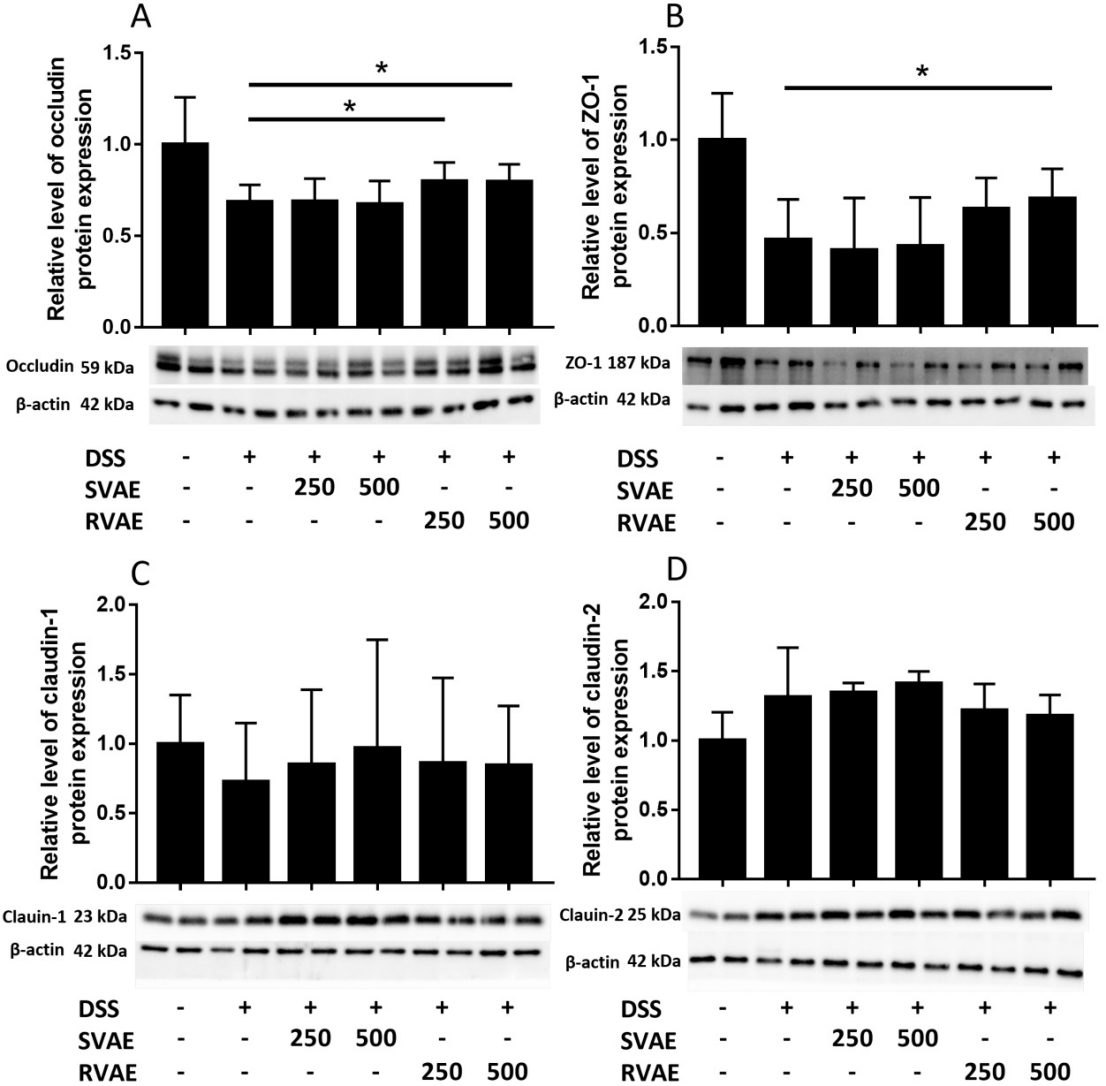
Effects of VA water extracts on tight junction protein in (A) occludin (B) Zonula occludens-1 (ZO-1) (C) claudin-1 and (D) claudin-2 of Caco-2 cells. Values are mean ± SD (n = 8). * Significantly difference (*P* < 0.05) are compared with the DSS treated group as negative control. The samples at the dosages of 250 μg mL^-1^ and 500 μg mL^-1^ of SVAE and RVAE are co-cultured until formation of the monolayer. The cells are collected and analyzed by immunoblotting.

Maintenance of TEER value and reduced permeability represented better barrier function of Caco-2 monolayer. We further investigated the alteration of tight junction proteins which were associated in barrier integrity. Immunoblot analyses showed that the expression of ZO-1, occludin, and claudin-1 markedly decreased in the DSS-treated group compared to the control group. However, treatment with RVAE significantly promoted the expression of ZO-1 and occludin to against DSS damage (*P* < 0.05). In claudin-1 expression of both SVAE and RVAE group tended to be higher than the DSS-treated group (Figure 3). DSS severely impaired the tight junction proteins while pretreatment with VA water extracts was able to improve protein expression, which might be the possible mechanism to keep the Caco-2 barrier function.

### Velvet antler water extracts upregulated the expression of myosin light chain kinase (MLCK) of Caco-2 cells

**Figure 4:**
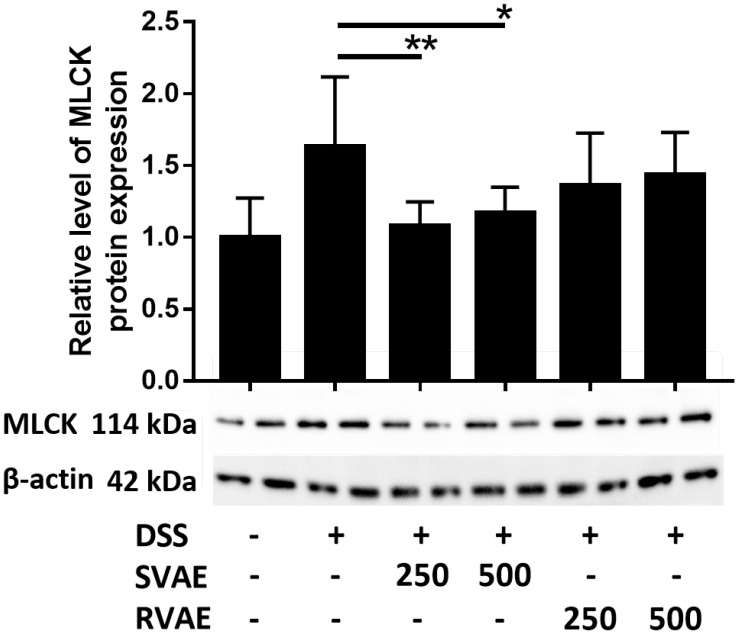
Effects of VA water extracts on myosin light chain kinase of Caco-2 cells. Values are mean ± SD (n = 8). * Significantly difference (*P* < 0.05) are compared with the DSS treated group as negative control. The samples at the dosages of 250 μg mL^-1^ and 500 μg mL^-1^ of SVAE and RVAE are co-cultured until formation of monolayer. The cells are collected and analyzed by immunoblotting. DSS: dextran sodium sulfate. SVAE: Sambar velvet antler water extracts. RVAE: red velvet antler water extracts.

To clarify the possible pathway of the expression of epithelium junction proteins, immunoblotting of MLCK, an integral biomarker of activation the cytoskeleton to further alter the tight junction proteins was performed. In contract to the result of those tight junction protein, MLCK expression in DSS treated group dramatically increased during barrier impairment. However, in the group of both SVAE and RVAE, the expression of MLCK was diminished. Expression of protein in SVAE group showed significant difference (*P* < 0.05) as compared with DSS treated group, which might be the possible mechanism involved (Figure 4).

### Velvet antler water extracts elevated the CCL20 production

**Figure 5:**
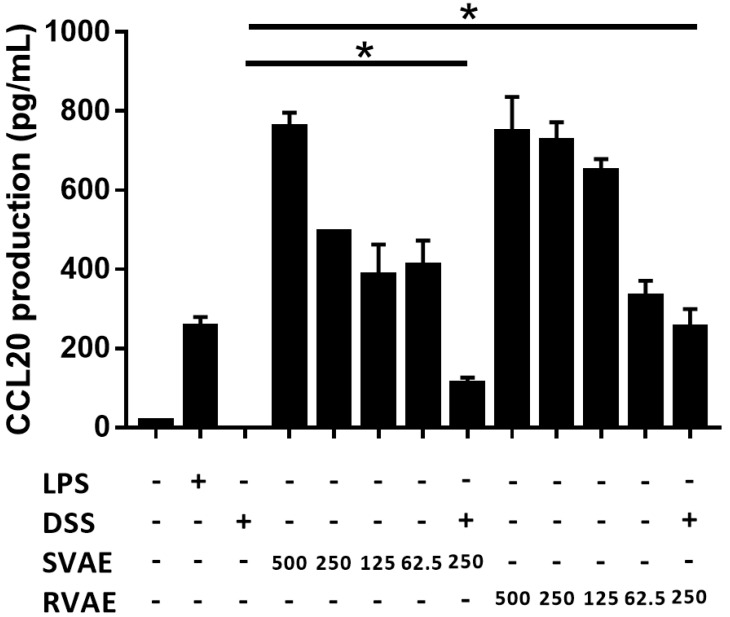
Effects of different VA water extracts on CCL20 production of Caco-2 cells. Values are mean ± SD (n = 3). * Significantly difference (*P* < 0.05) are compared with the DSS treated group as negative control. The samples at the tested dosages of VA water extracts are co-cultured for 24 hours. After treatment, the supernatant was collected and measured by ELISA assay. MLCK: myosin light chain kinase. DSS: dextran sodium sulfate. SVAE: Sambar velvet antler water extracts. RVAE: red velvet antler water extracts.

CCL20, involving in the restitution of colonic epithelial cells, is a crucial marker. Results indicated that production of CCL20 at tested dosage from 62.5 μg mL^-1^ to 500 μg mL^-1^ of VA water extracts culturing was significantly higher than the DSS treating group in a dose-dependent manner (*P* < 0.05). The results suggested that the velvet antler water extracts against DSS damage *in vitro* might involve the induction of the immune response to repair the barrier function (Figure 5).

## Discussion

Velvet antler has been widely used as folk medicine in Asian countries for a long time, however, there are fewer related studies on the functionalities of the Taiwan indigenous species, Formosan Sambar deer. Previous researches of velvet antler water extracts were focused on their anti-inflammatory activity and wound healing effects. Our investigation is the first study to evaluate the effects of VA extracts on the barrier function of intestinal epithelial cells *in vitro*. In the present study, we first demonstrated that pre-treated with the 250 μg mL^-1^ SVAE and RVAE with Caco-2 cells could augment the epithelial barrier function to against DSS challenge by improving TEER value and permeability. In addition, the cytotoxicity assay showed that co-cultured with VA extracts exhibited better cell viability of Caco-2 cells. These findings implied that the VA extracts of Formosan Sambar deer and red deer might be the candidates on amelioration of intestinal epithelial damage.

We further investigated the potential mechanisms involving anti-colitis effect. Previous studies have suggested that tight junction proteins, including occludin and claudin family, would be the highly related to the intestinal permeability and mucus integrity of IBD 33. The protective effect of VA extracts may be through the alteration of tight junction proteins expression. Significantly higher protein expression of occludin and ZO-1 with RVAE treatment might strengthen the barrier function of Caco-2 cell monolayer. Conversely, the claudin-2 showed the completely opposite trend, which was consistent with the clinical observation of patients with IBD 34, 35. Moreover, ZO-1 and claudin-2 proteins apparently involved the intracellular signal transduction pathways of MLCK and F-actin in dynamic homeostasis 36, 37. In this study, SVAE significantly downregulated the expression level of MLCK; while RVAE did not affect the MLCK expression. In addition to the expression of tight junction proteins, certain pro-inflammatory cytokines such as TNF-α, Interleukin 1β (IL-1β) and IFN-γ were able to activate the expression of MLCK 38, 39. Since MLCK is the limiting enzyme of activation with redistribution of cytoskeleton morphologically, anti-MLCK agents might be a potential candidate for treatment of inflammatory diseases, including atherosclerosis, pancreatitis and IBD 40. Our results indicated that RVAE and SVAE may possess the anti-IBD components that should be further purified and identified. Besides, both SVAE and RVAE significantly upregulated the production of CCL20 in a dose dependent manner. CCL20 would mobilize the intracellular calcium to evoke reorganization of the actin cytoskeleton, which contributed to the mucosal healing process and epithelial cell migration 41.

In summary, we concluded that both SVAE and RVAE possessed intestinal protection effects. The underlying mechanism appear to affect the intestinal barrier function, including promoting intestinal epithelial cell proliferation, improving the expression of occludin and ZO-1 probably *via* downregulating the MLCK activity and elevating the production of CCL20 chemokine to augment the barrier function and maintain the monolayer integrity to against from the DSS damage. Future research will focus on the exactly mechanism in animal study.

## Abbreviation

VA: Velvet antler
SVAE: Formosan Sambar deer water extracts
RVAE: Red deer water extracts
DSS: Dextran sulfate sodium
Caco-2: Colonic epithelial cell model
TEER: Trans-epithelial electrical resistance
ZO-1: Zonula occludens-1
MLCK: Myosin light chain kinase
CCL20: C-C chemokine ligand 20
IBD: Inflammatory bowel disease
TNF-α: Tumor necrosis factor-α
IFN-γ: Interferon-γ
IL-6: Interleukin-6
IL-1β: Interleukin 1β
FITC: Fluorescein isothiocyanate)
MTT: 3-(4,5-Dimethylthiazol-2-yl)-2,5-diphenyltetrazolium bromide
DMSO: Dimethyl sulfoxide
LPS: Lipopolysaccharide
